# The complete chloroplast genome of *Sargassum horneri* and its phylogenetic analysis

**DOI:** 10.1080/23802359.2019.1673237

**Published:** 2019-10-07

**Authors:** Yutong Cui, Tao Liu, Xumin Wang, Jiangyong Qu, Xuli Jia

**Affiliations:** a College of Life Sciences, Yantai University, Yantai, Shandong, People’s Republic of China;; b Hainan Academy of Ocean and Fisheries Sciences, Haikou, Hainan, People’s Republic of China;; c Hainan Provincial Key Laboratory of Technology for Tropical Seawater Aquaculture, Haikou, Hainan, People’s Republic of China;; d Laboratory of Genetics and Breeding of Marine Organism, College of Marine Life Sciences, Ocean University of China, Qingdao, Shandong, People’s Republic of China

**Keywords:** *Sargassum horneri*, phylogenetic analysis, chloroplast genome

## Abstract

Complete chloroplast genome of *Sargassum horneri* was obtained in this work. Circular mapping revealed that the complete chloroplast DNA sequences of *S. horneri* was 124,075 bp in length and had an overall AT content of 69.41%, including 139 protein-coding genes, 28 transfer RNA genes, and 6 ribosomal RNA genes. The phylogenetic tree shows that *S. horneri* and *Sargassum confusum* constituted a sister clade along with *Fucus vesiculosus*.

*Sargassum horneri*, belongs to the class of Phaeophyceae (Cho et al. 2006), is an economically important macroalgal in *Sargassum* genus that is highly valued for use in foodstuffs and industrial raw materials of extractions of iodine, algin, mannitol among others (Murakami et al. 2011). There are cultivation and application research in China. However, the phenomenon of *S. horneri* explosive growth and accumulating at coastal waters is called ‘golden tides’ (Smetacek and Zingone 2013), which causes detrimental effects on aquaculture, fisheries and the coastal ecosystems (Byeon et al. 2019). In this study, we obtained the complete chloroplast genome of *S. horneri*. Compared with the existing sequence (GenBank accession number NC029856) on NCBI, our sequence is 7 bp longer, with three base fragment variations in the intergenic region. The specimen was collected from Chi’ao, Fujian Province, China (26°33′42.82″N, 119°55′46.13″E), and stored at the Culture Collection of Seaweed at the Ocean University of China with an accession number 2018040199. Total DNA was extracted using the modified CTAB method (Doyle and Doyle 1990). Paired-end reads were sequenced using Illumina HiSeq × Ten system (Illumina, San Diego, CA). The experimental methods and data analysis were followed by the previous reports (Tamura et al. 2013; Liu et al. 2019).

The complete chloroplast genome of *S. horneri* is a circular DNA molecule measuring 124,075 bp in length with the overall A + T content of 69.41% (GenBank accession number MN265366). The nucleotide composition was 34.54% A (42,858 bp), 15.71% G (19,486 bp), 34.87% T (43,264 bp), and 14.88% C (18,467 bp). The chloroplast genome of *S. horneri* encoded 173 genes, which contained 137 protein-coding genes, 2 open reading frames, 28 transfer RNA genes, and 6 ribosomal RNA genes. All genes show the typical gene arrangement conforming to the Phaeosporeae consensus. Excepting *psbF* gene, which uses the typical GTG as the start codon, the rest protein-coding genes had the typical initiation ATG as the start codon. About 79.86% protein-coding genes had TAA as termination codon and the remaining were TAG (17.99%) and TGA (2.16%). There were 5 pairs of genes with overlapping by 4–53 bp, making full use of nucleotide and genetic information. All transfer RNA genes have the typical cloverleaf structure ranged from 71–293 bp.

All Phaeophyceae species with complete chloroplast genome available in the GenBank were selected to construct the phylogenetic tree by the Bayesian method, with *Phaeodactylum tricornutum* (Bacillariophyceae) as an outgroup. Phylogenetic analysis based on combined 30 protein-encoding genes (*psbI*, *atpA*, *psaA*, *petA*, *psaM*, *chlI*, *dnaK*, *rpoA*, *psbV*, *secA*, *rpoC2*, *atpG*, *rpl35*, *rbcS*, *psbA*, *ycf3*, *psaJ*, *ycf4*, *psaI*, *rps16*, *ycf39*, *dnaB*, *psbB*, *psaE*, *rpl31*, *rpl36*, *rps8*, *rpl29*, *rpl2*, and *rpl21*) exhibited that all the species of Phaeophyceae firstly clustered together and divided into two branches, *S. horneri* and *Sargassum confusum* constituted a sister clade along with *Fucus vesiculosus* (Figure 1). Results support current taxonomic systems.

**Figure 1.**
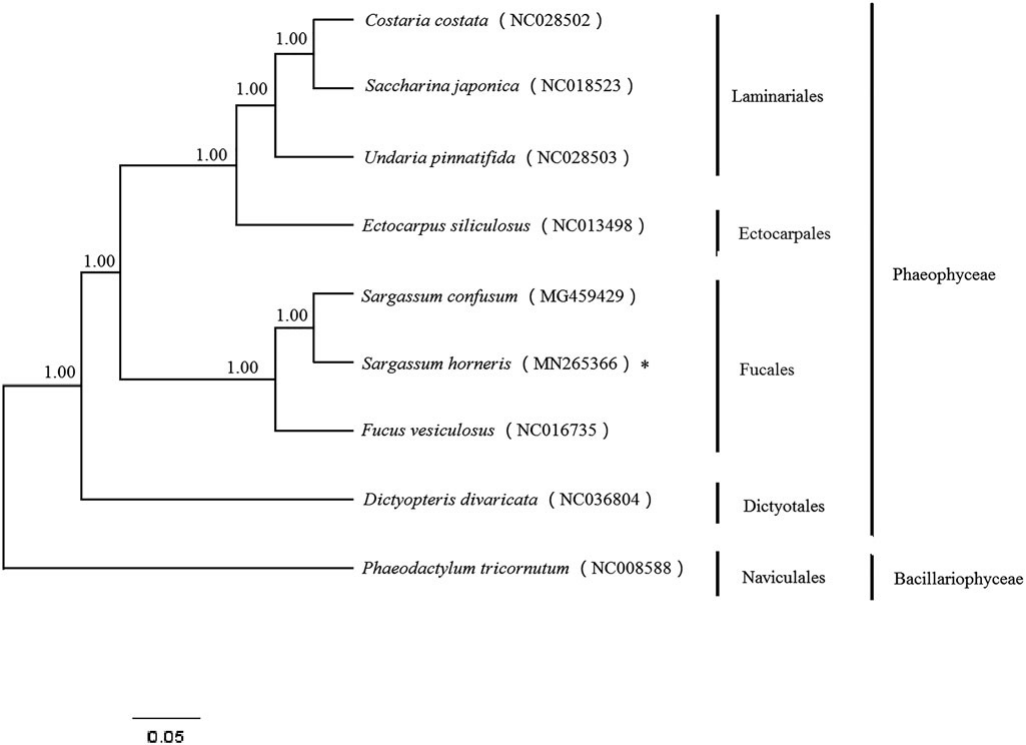
Phylogenetic tree (Bayesian inference) based on complete chloroplast genome of Phaeophyceae. Support values for each node were calculated from Bayesian posterior probability (BPP). Asterisks following species names indicate newly determined chloroplast genomes.
